# Association between Volatile Organic Compound Exposure and Sex Hormones in Adolescents: The Mediating Role of Serum Albumin

**DOI:** 10.3390/toxics12060438

**Published:** 2024-06-18

**Authors:** Xinyao Lian, Jianhui Guo, Yaqi Wang, Shaoguan Wang, Jing Li

**Affiliations:** Institute of Child and Adolescent Health, School of Public Health, Peking University, Beijing 100191, China; a987268196@163.com (X.L.); gjh201001@163.com (J.G.); 15878641416@163.com (S.W.)

**Keywords:** volatile organic compounds, sex hormones, adolescent, serum albumin

## Abstract

The associations between VOCs and sex hormones in adolescents remain unclear, and the role of serum albumin in these associations deserves to be explored. We conducted cross-sectional analyses using generalized linear models (GLMs), weighted quantile sum (WQS) regression, and mediation analysis, based on data from 584 adolescents from the National Health and Nutrition Examination Survey (NHANES). The GLM analyses revealed that seven kinds of mVOCs potentially affected sex hormone levels. According to the WQS regression results, 2-aminothiazoline-4-carboxylic acid (ATCA) was the major contributor to the significant associations of mixed mVOC exposure with testosterone, estradiol, and free androgen index in males; N-acetyl-S-(N-methylcarbamoyl)-L-cysteine (AMCC) was the major contributor to the significant associations of mixed mVOC exposure with sex hormone-binding globulin in males; and N-acetyl-S-(benzyl)-L-cysteine (BMA) was the major contributor to the significant associations of mixed mVOC exposure with the ratio of testosterone to estradiol in females. Moreover, serum albumin could mediate up to 9.2% of the associations between mixed exposure to mVOCs and sex hormones. Our findings could provide a reference for studies on the mechanisms underlying the effects of VOCs on sex hormones in adolescents and emphasize the necessity of reducing exposure to ATCA, AMCC, BMA, and their parent compounds.

## 1. Introduction

Sex hormones play an important role in promoting the development of secondary sex characteristics and influencing the reproductive system during puberty [1]. Also, sex hormones affect several systems, including the skeletal, immune, and nervous systems [2,3]. Abnormal changes in sex hormones may lead to delayed puberty, polycystic ovary syndrome, increased risk of cardiovascular disease, and other health problems [1,4]. Adolescents may be vulnerable to some volatile chemical products, such as methylbenzene, which may cause changes in sex hormone levels due to the inefficiency of their detoxification systems [5,6].

Volatile organic compounds (VOCs) come from a wide range of sources, which include outdoor sources, such as natural emissions, vehicle emissions, solvent use, and biomass combustion, and indoor sources, such as furniture, laminate wood flooring, decorative materials made of wood-based panels, glues, and other daily necessities [7,8,9,10]. With their high volatility, wide range of sources, and multiple routes of exposure, VOCs are hazardous to human health and deserve more attention [7,8]. Possible associations of VOCs with leukemia and cardiovascular and respiratory diseases have been demonstrated [11]. In particular, several VOCs have been proven to have endocrine-disrupting properties, which may lead to a decrease in the levels of thyroid-stimulating hormones and disruption of pituitary hypothalamic–adrenocortical activity [6,12]. Growing research is focusing on the impact of VOCs on reproductive health. Animal studies revealed that VOC exposure may damage testicular structure, affecting sperm cell counts and sperm viability [13]. Epidemiological studies also showed that VOCs could adversely impact the structure and function of the female reproductive system, as well as the sperm DNA of males [14,15]. Studies in populations of adult males and females in the United States revealed that specific VOC exposures were risk factors for sex hormone disruption and that 2,5-dimethylfuran had a greater effect [16,17]. Differences in VOC exposure patterns between adolescents and adults may lead to differences in the effects of VOCs between the adolescent and adult populations [18]. Additionally, a study showed that adolescents had higher levels of VOC metabolites than adults, possibly because their ratio of lung surface area to body weight and their respiratory rates were higher than those of adults [19,20]. However, associations between exposure to VOCs and sex hormone changes in adolescents remain unclear.

Serum albumin might play an important role in the effects of VOCs on sex hormones. Studies have confirmed that serum albumin may mediate the relationship between several endocrine-disrupting chemicals and sex hormones [21]. In addition, increased concentrations of metabolites of VOCs, including benzene, could lead to structural changes in serum albumin [22], which might influence the regulation of sex hormones by serum albumin [23]. Exploring the role of serum albumin in the associations between VOCs and sex hormones could support the mechanistic exploration of the effects of VOCs on sex hormones, but studies on the mediating role of serum albumin were lacking.

To fill this research gap, we assessed the associations of single and mixed metabolites of VOCs (mVOCs) with sex hormones and the role of serum albumin in these associations based on the National Health and Nutrition Examination Survey (NHANES) data.

## 2. Methods

### 2.1. Study Population

The NHANES is a nationwide cross-sectional survey conducted under the guidance of the National Center for Health Statistics (NCHS) and the Centers for Disease Control and Prevention (CDC) to assess the health and nutritional status of adults and children in the United States. Participants underwent health interviews at home and received physical examinations and the collection of urine and blood samples at specially designed and equipped mobile centers. The study procedures were approved by the NCHS Ethics Review Board, and written informed consents were obtained from all participants [24].

**Figure 1:**
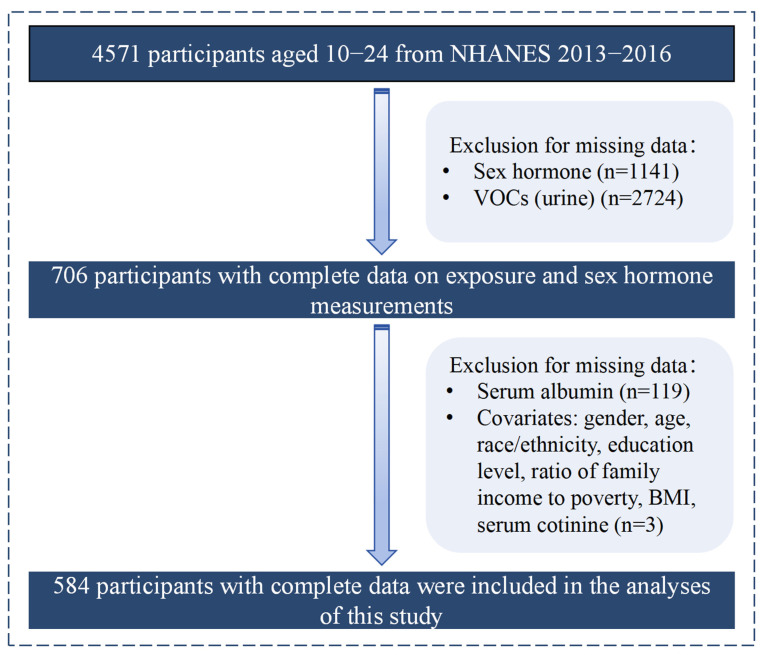
Flowchart of studied participant selection (N = 584) from the NHANES, 2013–2016. Notes: VOCs: volatile organic compounds; BMI: body mass index.

We utilized publicly available data from two cycles (2013–2014 and 2015–2016) from the NHANES. Participants whose data were collected before 2013 were excluded due to the lack of complete sex hormone data. A total of 584 adolescents with complete survey data on selected urinary mVOCs, serum sex hormones, serum albumin, and selected covariates were included in this study. The participant screening process is shown in Figure 1.

### 2.2. Sex Hormone Measurement

Total testosterone (TT) and estradiol (E_2_) in serum were estimated with isotope dilution liquid chromatography tandem mass spectrometry. As a blood transport protein for androgens and estrogens, sex hormone-binding globulin (SHBG) is measured indirectly using chemiluminescent measurements of photomultiplier tubes to measure the reaction products from the reaction of SHBG with immune antibodies. The analytical methodology was described explicitly in the Laboratory Procedures Handbook on the NHANES website [25]. Circulating free testosterone was assessed indirectly through the free androgen index (FAI) and the ratio of testosterone to estradiol (TT/E_2_). The FAI was calculated with the following formula: [(testosterone × 100)/SHBG] [26].

### 2.3. VOC Measurement

Compared to parent compounds, urine mVOCs have longer physiological half-lives and can effectively assess exposure to VOCs [27]. Ultra-performance liquid chromatography coupled with electrospray tandem mass spectrometry (UPLC-ESI/MSMS) was used to measure the human urine mVOCs [28]. In particular, for mVOCs with analysis results below the lower limit of detection, the lower limit of detection divided by the square root of 2 was used as the interpolation fill value. Detailed information related to the measurements can be found elsewhere [28]. To make the results more reliable and meaningful, we chose to include mVOCs with a detection rate higher than 50%. A total of 17 mVOCs were included in this study, and the specific species assessed are shown in Table S1. The urinary creatinine levels were used to adjust all urinary mVOC concentrations to avoid measurement errors due to external causes such as metabolic levels or water intake [29].

### 2.4. Covariates

Based on previous studies [16,17,21], a directed acyclic graph (DAG) approach was applied to select potential adjustment variables [30], which included sex, age, race/ethnicity, education level, body mass index (BMI), serum cotinine concentration, the ratio of family income to poverty, and serum albumin. DAG (R packages “dagitty” and “ggdag”) showed the main relationships between exposure, outcome, covariates, and the mediator (Figure S1).

### 2.5. Statistical Analysis

Given their skewed distributions, the urinary mVOCs and sex hormones were log10-transformed to improve the normality of the regression analyses. Pearson correlation coefficients were calculated with the transformed mVOC concentrations to assess the correlation between the mVOCs. We utilized generalized linear models (GLMs) to evaluate the association between each urinary mVOC and each sex hormone, taking the transformed concentration of each mVOC as the continuous exposure variable and the transformed values of each sex hormone as the continuous outcome variable. The regression coefficient (β) was interpreted as the average change in the transformed sex hormone for each unit increase in each transformed mVOC.

We used weighted quantile sum (WQS) regression to estimate the associations between mixed exposures to urinary mVOCs and sex hormones. The WQS has been widely used to assess the effects of mixed exposures to multiple environmental pollutants [31]. It allowed for covariance and could identify the contribution of individual chemicals that led to the observed associations [26]. We constructed regression coefficients (β) for the WQS index of mVOCs to demonstrate the average change in transformed sex hormones per unit increase in the index. We calculated the mixed exposure concentrations based on the weights of the mVOCs in the WQS models. In addition, a mediation analysis was performed to explore the role of serum albumin in the association between mixed exposure to mVOCs and sex hormones.

Sensitivity analyses were conducted with the Bayesian kernel machine regression (BKMR) model. The joint effects of multiple pollutants were assessed using the change in sex hormones when all chemicals were fixed at the 75th percentile, compared to those fixed at the 25th percentile. Conditional posterior inclusion probabilities (PIPs) calculated in the BKMR models were used to estimate the relative importance of individual exposure variables to the overall mixture effects [32].

We used sex stratification to explore the associations between exposure to mVOCs and sex hormones under different sexes, and comparisons of associations by sex were performed with the 2-sample z-test. The Benjamini–Hochberg (BH) procedure was used to adjust for multiple comparisons [33]. All analyses in this study were performed in R version 4.3.2. The R packages “wqs”, “bkmr”, and “mediation” were used for WQS analysis, BKMR analysis, and mediation analysis, respectively. Two-sided *p*-values < 0.05 indicated statistical significance.

## 3. Results

### 3.1. Description of General Characteristics

A total of 584 participants with an average age of 17.01 ± 3.70 years were included. The age range of participants after screening was 12–24 years. Among the participants, 51.20% were male, 26.54% were non-Hispanic White, and 35.27% had an education below the ninth grade level. In addition, the average BMI was 24.92 ± 6.37 kg/m^2^, the mean ratio of family income to poverty was 2.07 ± 1.48, the average level of serum cotinine was 16.46 ± 62.16 ng/mL, and the average level of serum albumin was 4.49 ± 0.29 g/dL. The specific characteristics of the participants are presented in Table 1.

The detection limits and distribution characteristics of urinary mVOCs, testosterone, estradiol, SHBG, FAI, and TT/E_2_ in the study population are displayed in Table S2. As shown in Figure S2, there were significant correlations between urinary mVOCs, with Pearson correlation coefficients ranging from −0.03 to 0.81.

### 3.2. Associations of Single Exposure to mVOCs and Serum Albumin Exposure with Sex Hormones

**Figure 2:**
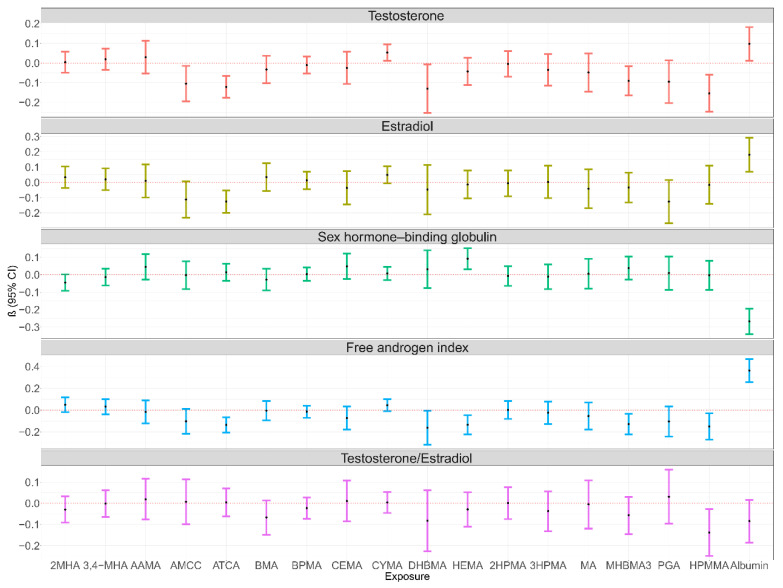
Association of single exposure to mVOCs and serum albumin exposure with sex hormones in the NHANES, 2013–2016 (N = 584). Notes: 2MHA: urinary 2-Methylhippuric acid; 3,4-MHA: urinary 3- and 4-Methylhippuric acid; AAMA: urinary N-Acetyl-S-(2-carbamoylethyl)-L-cysteine; AMCC: urinary N-Acetyl-S-(N-methylcarbamoyl)-L-cysteine; ATCA: urinary 2-Aminothiazoline-4-carboxylic acid; BMA: urinary N-Acetyl-S-(benzyl)-L-cysteine; BPMA: urinary N-Acetyl-S-(n-propyl)-L-cysteine; CEMA: urinary N-Acetyl-S-(2-carboxyethyl)-L-cysteine; CYMA: urinary N-Acetyl-S-(2-cyanoethyl)-L-cysteine; DHBMA: urinary N-Acetyl-S-(3,4-dihydroxybutyl)-L-cysteine; HEMA: urinary N-Acetyl-S-(2-hydroxyethyl)-L-cysteine; 2HPMA: urinary N-Acetyl-S-(2-hydroxypropyl)-L-cysteine; 3HPMA: urinary N-Acetyl-S-(3-hydroxypropyl)-L-cysteine; MA: urinary Mandelic acid; MHBMA3: urinary N-Acetyl-S-(4-hydroxy-2-butenyl)-L-cysteine; PGA: urinary Phenylglyoxylic acid; and HPMMA: urinary N-Acetyl-S-(3-hydroxypropyl-1-methyl)-L-cysteine. Adjusted for sex, age, race/ethnicity, education level, BMI, serum cotinine concentration, and the ratio of family income to poverty.

The results of the GLM analyses showed that N-acetyl-S-(N-methylcarbamoyl)-L-cysteine (AMCC), 2-aminothiazoline-4-carboxylic acid (ATCA), N-acetyl-S-(3,4-dihydroxybutyl)-L-cysteine (DHBMA), N-acetyl-S-(4-hydroxy-2-butenyl)-L-cysteine (MHBMA3), and N-acetyl-S-(3-hydroxypropyl-1-methyl)-L-cysteine (HPMMA) were negatively correlated with testosterone, and N-acetyl-S-(2-cyanoethyl)-L-cysteine (CYMA) and serum albumin were positively correlated with testosterone, after adjusting for covariates, including sex, age, race/ethnicity, education level, BMI, serum cotinine concentration, and the ratio of family income to poverty (Figure 2). For estradiol, ATCA was negatively correlated with estradiol_,_ and serum albumin was positively correlated with estradiol. For SHBG, N-acetyl-S-(2-hydroxyethyl)-L-cysteine (HEMA) was positively correlated with SHBG, and serum albumin was negatively correlated with SHBG. For the FAI, ATCA, DHBMA, HEMA, MHBMA3, and HPMMA were negatively correlated with the FAI, and serum albumin was positively correlated with the FAI. In addition, HPMMA was negatively correlated with TT/E_2_. Similar associations were found between mVOCs, serum albumin, and sex hormones stratified by sex (Figures S3 and S4).

### 3.3. Associations between Mixed Exposure to mVOCs and Sex Hormones

According to the results of the WQS analyses, mixed exposure to mVOCs was significantly negatively associated with testosterone, estradiol, FAI, and TT/E_2_ (Table 2). For the male group, mixed exposure to mVOCs was significantly negatively associated with testosterone, estradiol, and FAI, while it was positively associated with SHBG. For the female group, there was a significant negative association between mixed exposure to mVOCs and TT/E_2_. Differences were found in the associations between mixed exposure to mVOCs and sex hormone indicators by sex.

**Figure 3:**
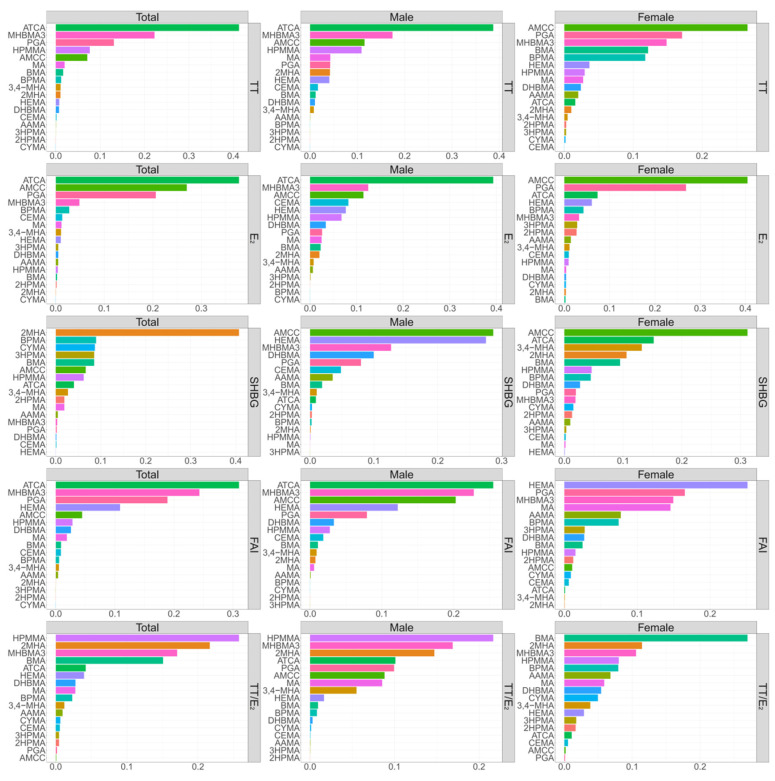
Weights for mVOCs associated with sex hormones from the WQS regression model. Notes: WQS: weighted quantile sum; 2MHA: urinary 2-Methylhippuric acid; 3,4-MHA: urinary 3- and 4-Methylhippuric acid; AAMA: urinary N-Acetyl-S-(2-carbamoylethyl)-L-cysteine; AMCC: urinary N-Acetyl-S-(N-methylcarbamoyl)-L-cysteine; ATCA: urinary 2-Aminothiazoline-4-carboxylic acid; BMA: urinary N-Acetyl-S-(benzyl)-L-cysteine; BPMA: urinary N-Acetyl-S-(n-propyl)-L-cysteine; CEMA: urinary N-Acetyl-S-(2-carboxyethyl)-L-cysteine; CYMA: urinary N-Acetyl-S-(2-cyanoethyl)-L-cysteine; DHBMA: urinary N-Acetyl-S-(3,4-dihydroxybutyl)-L-cysteine; HEMA: urinary N-Acetyl-S-(2-hydroxyethyl)-L-cysteine; 2HPMA: urinary N-Acetyl-S-(2-hydroxypropyl)-L-cysteine; 3HPMA: urinary N-Acetyl-S-(3-hydroxypropyl)-L-cysteine; MA: urinary Mandelic acid; MHBMA3: urinary N-Acetyl-S-(4-hydroxy-2-butenyl)-L-cysteine; PGA: urinary Phenylglyoxylic acid; HPMMA: urinary N-Acetyl-S-(3-hydroxypropyl-1-methyl)-L-cysteine; TT: testosterone; E_2_: estradiol; SHBG: sex hormone-binding globulin; FAI: free androgen index; and TT/E_2_: ratio of testosterone to estradiol.

Furthermore, we found that ATCA was the main mVOC affecting testosterone, estradiol, and FAI levels in the male population, while AMCC had a major effect on SHBG levels (Figure 3). In females, BMA was the major metabolite affecting TT/E_2_.

We conducted sensitivity analyses with the BKMR model and found that the associations between mixed exposure to mVOCs and sex hormones were similar to the results obtained with the WQS model (Figure S5 and Table S3). For males, mixed exposure to mVOCs was negatively associated with testosterone, estradiol, and FAI, and ATCA was the main mVOC affecting testosterone, estradiol, and FAI. For females, the associations of mixed mVOC exposure with most of the sex hormone indicators were not significant.

### 3.4. Mediation Analyses

**Figure 4:**
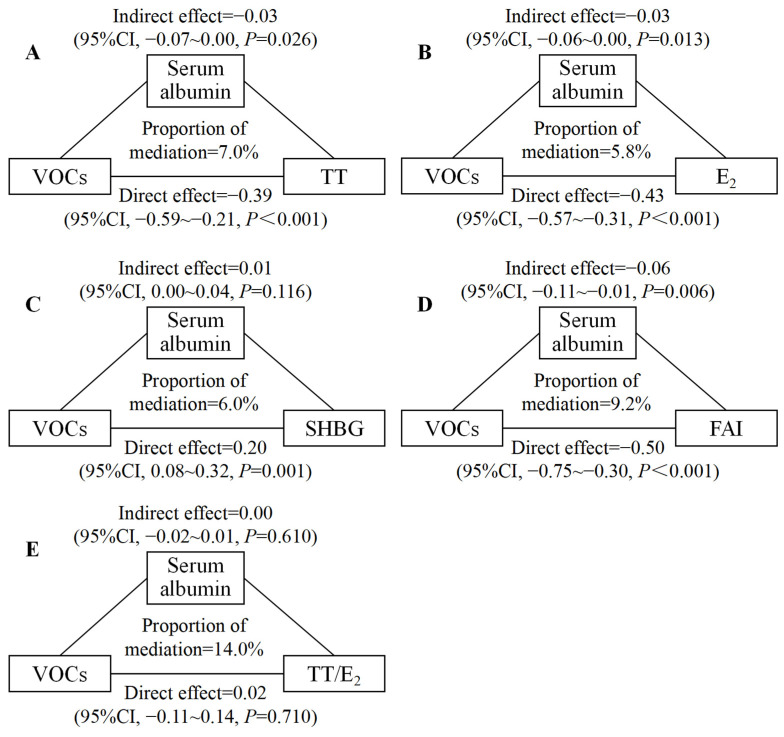
Estimated proportions of associations between mixed exposure to mVOCs and TT, E_2_, SHBG, FAI, and TT/E_2_ mediated by serum albumin in males (N = 299). Adjusted for age, race/ethnicity, education level, BMI, serum cotinine concentration, and the ratio of family income to poverty. (**A**) Estimated proportions of associations between mixed exposure to mVOCs and TT mediated by serum albumin in males; (**B**) Estimated proportions of associations between mixed exposure to mVOCs and E_2_ mediated by serum albumin in males; (**C**) Estimated proportions of associations between mixed exposure to mVOCs and SHBG mediated by serum albumin in males; (**D**) Estimated proportions of associations between mixed exposure to mVOCs and FAI mediated by serum albumin in males; (**E**) Estimated proportions of associations between mixed exposure to mVOCs and TT/E_2_ mediated by serum albumin in males. Notes: CI: confidence interval; VOCs: volatile organic compounds; TT: testosterone; E_2_: estradiol; SHBG: sex hormone-binding globulin; FAI: free androgen index; and TT/E_2_: ratio of testosterone to estradiol.

**Figure 5:**
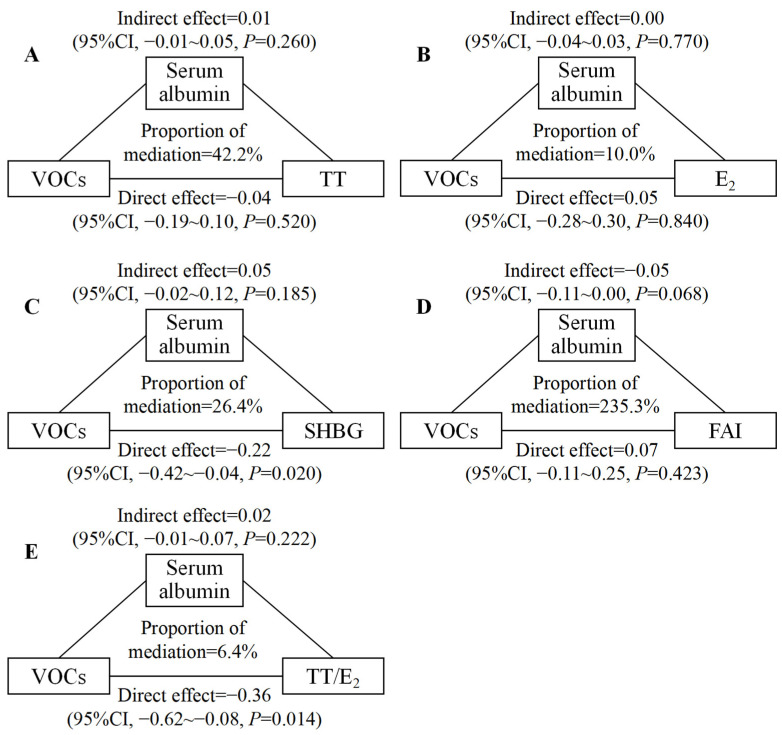
Estimated proportions of associations between mixed exposure to mVOCs and TT, E_2_, SHBG, FAI, and TT/E_2_ mediated by serum albumin in females (N = 285). Adjusted for age, race/ethnicity, education level, BMI, serum cotinine concentration, and the ratio of family income to poverty. (**A**) Estimated proportions of associations between mixed exposure to mVOCs and TT mediated by serum albumin in females; (**B**) Estimated proportions of associations between mixed exposure to mVOCs and E_2_ mediated by serum albumin in females; (**C**) Estimated proportions of associations between mixed exposure to mVOCs and SHBG mediated by serum albumin in females; (**D**) Estimated proportions of associations between mixed exposure to mVOCs and FAI mediated by serum albumin in females; (**E**) Estimated proportions of associations between mixed exposure to mVOCs and TT/E_2_ mediated by serum albumin in females. Notes: CI: confidence interval; VOCs: volatile organic compounds; TT: testosterone; E_2_: estradiol; SHBG: sex hormone-binding globulin; FAI: free androgen index; and TT/E_2_: ratio of testosterone to estradiol.

Our mediation analyses showed that serum albumin mediated 7.0% of the association between mixed exposure to mVOCs and testosterone, 5.8% of the association between mixed exposure to mVOCs and estradiol, and 9.2% of the association between mixed exposure to mVOCs and FAI, respectively, in males (Figure 4). We observed no significant mediating effect of serum albumin in females (Figure 5). The mediating effect of serum albumin on the associations between mixed exposure to mVOCs and sex hormones in total participants is shown in Figure S6.

## 4. Discussion

This study analyzed the complex associations between urinary mVOCs and sex hormones among adolescents aged 12–24 in the United States from 2013 to 2016. We found that AMCC, ATCA, CYMA, DHBMA, MHBMA3, HPMMA, and HEMA might influence sex hormone levels. Mixed exposure to mVOCs was associated with testosterone, estradiol, FAI, and SHBG levels in males, with ATCA significantly associated with testosterone, estradiol, and FAI and AMCC significantly associated with SHBG. In the female population, mixed exposure to mVOCs was associated with TT/E_2_, with BMA being the most influential factor. Moreover, we found that serum albumin mediated the associations of mVOCs with testosterone, estradiol, and FAI levels in males.

In the single exposure analyses, we found associations of AMCC, ATCA, CYMA, DHBMA, MHBMA3, and HPMMA with testosterone, ATCA with estradiol, HEMA with SHBG, ATCA, DHBMA, HEMA, MHBMA3, and HPMMA with FAI, and HPMMA with TT/E_2_. We suggested that N, N-dimethylformamide, cyanide, acrylonitrile, 1,3-butadiene, crotonaldehyde, vinyl chloride, and ethylene oxide were the parent compounds of AMCC, ATCA, CYMA, DHBMA, MHBMA3, HPMMA, and HEMA, respectively, and had the potential to influence sex hormones. Previous studies revealed that blood mVOCs were associated with sex hormones in adults [16,17]. N, N-Dimethylformamide affected the interaction of the estrogen receptor with estradiol, reduced fertility, increased offspring malformations, and could cause testicular germ cell tumors [34,35,36]. Crotonaldehyde might induce oxidative stress damage in the reproductive organs of male rats, affecting testicular enzyme function and hormone levels [37,38]. 1,3-Butadiene may be linked to ovarian atrophy, testicular atrophy, and male infertility [39]. Acrylonitrile, cyanide, vinyl chloride, and ethylene oxide were also recognized as being potentially toxic for reproduction [40,41,42,43,44,45]. These studies provided part of the support for our findings. In addition, our study filled a research gap on urinary mVOC exposure and sex hormones in adolescents and provided evidence for the effects of VOCs on sex hormones and on the reproductive system.

Considering that there were differences in both the levels and roles of sex hormones in the populations across sexes [46], we addressed the potential joint effects of mVOC exposure on sex hormones in adolescents stratified by sex. We found that mixed exposure to mVOCs was associated with most male sex hormone indicators but only associated with TT/E_2_ in females. Differences in lifestyle and other aspects between males and females could cause differences in the sources and species of VOC exposure, while differences in their physiological factors might cause differences in susceptibility to VOCs as well as differences in the uptake, transport, metabolism, storage, and excretion of VOCs, which could lead to differences in the impacts of VOCs in the different sex groups [47]. Additionally, a study of adult males indicated associations between mixed exposure to VOCs and sex hormones, which supported the hypothesis that mixed exposure to VOCs leads to sex hormone disruption as our study did, but the directions of the trends in associations with testosterone and estradiol were contrary to those of our study [17]. Differences in the results might be caused by differences in VOC exposure measurements and age groups of the study populations, and particularly, more drastic sex hormone changes in the adolescent population probably lead to differences in associations with VOCs [48]. Furthermore, we found important roles of AMCC, ATCA, and BMA in the associations between mixed exposure to mVOCs and sex hormones, showing that more attention should be paid to the health effects of their parent compounds, including N, N-dimethylformamide, cyanide, and toluene. N, N-Dimethylformamide was commonly used in the manufacture of films, fibers, paints, and polyurethane lacquers [36]; toluene was known to be involved in cosmetics, inks, adhesives, lacquers, and glues [49]; and cyanide was contained in rodenticides, pigments, and a variety of sources [50]. It was recommended to minimize the use of these substances in industrial products and household goods or reduce exposure to these sources.

The mechanism for the effects of VOCs on sex hormones is still unclear. As endocrine-disrupting chemicals, several VOCs might affect nuclear hormone receptors to influence sex hormone levels by participating in pathways involving ligand binding, receptor agonism or antagonism, receptor binding to transcriptional cofactors, DNA binding and differential gene expression, membrane-associated hormone receptor-mediated non-genomic signaling, and induction of epigenetic reprogramming [51,52]. Moreover, oxidative stress damage to gonadal tissues, effects on genes involved in steroidogenesis, inhibition of related enzymes, interference of the hypothalamic–pituitary–adrenal axis, and effects on serum albumin are the pathways by which endocrine-disrupting chemicals affect the production and transport of sex hormones, as well as possible mechanisms for the effects of VOCs on sex hormones [21,53,54,55,56,57]. 

We found that serum albumin served as a mediator in the associations of mVOCs with testosterone, estradiol, and FAI in males. Serum albumin is the most abundant protein in vertebrate plasma, binding and transporting a wide range of biologically active substances, which leads to its focus [58]. Some studies revealed the role of serum albumin as a mediator in the associations of endocrine-disrupting chemicals with sex hormones and lipids [21,59], but there were no studies before our study related to serum albumin mediating the relationships between VOCs and sex hormones. In particular, the effects of VOCs on serum albumin and the effects of serum albumin on sex hormones have been confirmed by research. VOCs can induce hepatic steatosis, metabolic disorders, oxidative stress, inflammation, and apoptosis, all of which contribute to liver damage [60]. This damage might affect the liver’s ability to produce serum albumin in its precursor form of pre-pro-albumin [58]. Additionally, VOCs can cause endoplasmic reticulum stress, which impairs the maturation process of serum albumin [58,60]. Consequently, these disruptions may lead to alterations in serum albumin concentrations. Moreover, VOCs probably cause changes in serum albumin concentrations by affecting C-reactive proteins, as well as structural changes in proteins, possibly by inducing oxidative stress [61,62,63]. Spectroscopic analysis also showed that the binding of VOCs to serum albumin altered the secondary structure of serum albumin [64]. Furthermore, serum albumin can contribute to the regulation of hormone levels by influencing the transport and activity of sex hormones in the body [65]. Albumin can also reduce the endocrine interference of exogenous substances, but alterations in serum albumin structure may bring about a reduction in function [66]. The research findings mentioned above might be helpful in explaining how serum albumin mediates the associations of VOCs with sex hormones.

Our study has several strengths. Firstly, our study was conducted based on nationally representative NHANES data from the U.S., which had a standardized study protocol, trained technicians, and quality control processes in the data collection process [26]. Secondly, our study filled the gap of studies on the associations between VOC exposure and sex hormone levels in adolescents and explored the role of serum albumin in these associations, which could provide mechanistic clues on the effects of VOCs on sex hormones and even the reproductive system. Moreover, our study used the WQS model and BKMR model to solve the multicollinearity problem and make the results more stable [31,67]. 

There remained some limitations to our study. First, the population in our study was relatively small and exclusively comprised U.S. adolescents, which may limit the extrapolation of the findings to populations in other regions. Further research is warranted to investigate the impact of VOCs on sex hormones in adolescents from different regions, despite our study being based on a nationally representative sample of the U.S. population. Second, our study utilized a cross-sectional design, and causal inference between mVOCs and sex hormones was limited. A further cohort study is needed to verify the causal associations of VOC exposure with sex hormone changes and the role of albumin in these associations. In addition, there were some confounders, including particulate matter, ozone, and road traffic noise, that may affect sex hormones [68,69,70]. However, we were unable to incorporate these factors into the model due to insufficient data or because they were not yet identified by us as confounders. More possible confounding factors should be considered in future studies to provide a more comprehensive analysis.

## 5. Conclusions

In conclusion, we found associations between seven kinds of mVOCs and sex hormone changes in adolescents, and these associations differed across sexes. In the male population, mixed exposure to mVOCs was associated with testosterone, estradiol, FAI, and SHBG, and serum albumin mediated the associations of mVOCs with testosterone, estradiol, and FAI. In females, mixed exposure to mVOCs was only associated with TT/E_2_ changes. We also found that AMCC, ATCA, and BMA played major roles in the associations between mixed exposure to mVOCs and sex hormones, which suggested we should reduce the exposure to their parent compounds, including N, N-dimethylformamide, cyanide, and toluene. This study might provide new insights into the mechanism of VOC exposure-induced endocrine disruption, which need to be further validated by cohort studies.

## Figures and Tables

**Table 1:** General characteristics of participants aged 10–24 years from the NHANES, 2013–2016 (N = 584).

Characteristics	Mean ± SD or n (%)
Age (year)	17.01 ± 3.7
Sex	
Male	299 (51.2)
Female	285 (48.8)
Race/ethnicity	
Mexican American	130 (22.26)
Other Hispanic	68 (11.64)
Non-Hispanic White	155 (26.54)
Non-Hispanic Black	127 (21.75)
Other Race	104 (17.81)
Education	
Less than 9th grade	206 (35.27)
9–11th grade	190 (32.53)
High school graduate/GED or equivalent	75 (12.84)
More than high school	113 (19.35)
BMI (kg/m^2^)	24.92 ± 6.37
Ratio of family income to poverty	2.07 ± 1.48
Serum cotinine (ng/mL)	16.46 ± 62.16
Serum albumin (g/dL)	4.49 ± 0.29

**Table 2:** Associations between the WQS index and sex hormone indicators in the NHANES, 2013–2016.

Variables	Testosterone	Estradiol	SHBG	FAI	TT/E_2_
Total	−0.104 (−0.137, −0.071) *	−0.096 (−0.139, −0.053) *	−0.012 (−0.043, 0.019)	−0.135 (−0.178, −0.092) *	−0.059 (−0.102, −0.016) *
Male	−0.181 (−0.232, −0.130) *	−0.185 (−0.224, −0.146) *	0.072 (0.039, 0.105) *	−0.234 (−0.295, −0.173) *	−0.008 (−0.043, 0.027)
Female	−0.026 (−0.069, 0.017)	−0.032 (−0.108, 0.044)	−0.054 (−0.109, 0.001)	−0.029 (−0.084, 0.026)	−0.126 (−0.206, −0.046) *

Notes: WQS: weighted quantile sum; SHBG: sex hormone-binding globulin; FAI: free androgen index; and TT/E_2_: ratio of testosterone to estradiol. *: *p* < 0.05.

## Data Availability

The original data presented in this study are openly available in the National Health and Nutrition Examination Survey at https://www.cdc.gov/nchs/nhanes/index.htm (accessed on 31 January 2024).

## Supplementary Materials

The following supporting information can be downloaded at: https://www.mdpi.com/article/10.3390/toxics12060438/s1, Figure S1: Directed acyclic graph; Figure S2: Pearson’s correlations between urinary mVOCs; Figure S3: Association of single exposure to mVOCs and serum albumin exposure with sex hormones among male populations in the NHANES, 2013–2016 (N = 299); Figure S4: Association of single exposure to mVOCs and serum albumin exposure with sex hormones among female populations in the NHANES, 2013–2016 (N = 285); Figure S5: The effects of mVOC mixture exposure on TT, E2, SHBG, FAI, and TT/E2 by the BKMR models; Figure S6: Estimated proportions of associations between mixed exposure to mVOCs and TT, E2, SHBG, FAI, and TT/E2 mediated by serum albumin (N = 584). Table S1: VOC metabolites, their abbreviations, and their parent compounds; Table S2: Detection limits and distribution of urinary mVOCs and sex hormones in the study population; Table S3: Posterior inclusion probabilities (PIPs) in the BKMR model in the NHANES, 2013–2016.
