# Supplementary material for: Association between Volatile Organic Compound Exposure and Sex Hormones in Adolescents: The Mediating Role of Serum Albumin
Source: Toxics. 2024 Jun 18;12(6):438. doi: 10.3390/toxics12060438 (PMC11209113; doi:10.3390/toxics12060438)
Supplement: Supplementary file 1 [file toxics-12-00438-s001.zip › toxics-3057766-supplementary.pdf]

Table S1. VOC metabolites, their abbreviations, and their parent compounds.

VOC metabolite	Common name	Parent compound
Urinary 2-Methylhippuric acid	2MHA	Xylene
Urinary 3- and 4-Methylhippuric acid	3,4-MHA	Xylene
Urinary N-Acetyl-S-(2-carbamoylethyl)-L-cysteine	AAMA	Acrylamide
Urinary N-Acetyl-S-(N-methylcarbamoyl)-L-cysteine	AMCC	N, N-Dimethylformamide
Urinary 2-Aminothiazoline-4-carboxylic acid	ATCA	Cyanide
Urinary N-Acetyl-S-(benzyl)-L-cysteine	BMA	Toluene
Urinary N-Acetyl-S-(n-propyl)-L-cysteine	BPMA	1-Bromopropane
Urinary N-Acetyl-S-(2-carboxyethyl)-L-cysteine	CEMA	Acrolein
Urinary N-Acetyl-S-(2-cyanoethyl)-L-cysteine	CYMA	Acrylonitrile
Urinary N-Acetyl-S-(3,4-dihydroxybutyl)-L-cysteine	DHBMA	1,3-Butadiene
Urinary N-Acetyl-S-(2-hydroxyethyl)-L-cysteine	HEMA	Acrylonitrile, vinyl chloride, and ethylene oxide
Urinary N-Acetyl-S-(2-hydroxypropyl)-L-cysteine	2HPMA	Propylene oxide
Urinary N-Acetyl-S-(3-hydroxypropyl)-L-cysteine	3HPMA	Acrolein
Urinary Mandelic acid	MA	Styrene
Urinary N-Acetyl-S-(4-hydroxy-2-butenyl)-L-cysteine	MHBMA3	1,3-Butadiene
Urinary Phenylglyoxylic acid	PGA	Ethylbenzene and styrene
Urinary N-Acetyl-S-(3-hydroxypropyl-1-methyl)-L-cysteine	HPMMA	Crotonaldehyde

Table S2. Detection limits and distribution of urinary mVOCs and sex hormones in the study population.

Variable	LLOD	10th percentile	25th percentile	50th percentile	75th percentile	90th percentile
2MHA (ng/mL)	5.000	-1.146	-0.916	-0.646	-0.298	-0.010
3,4-MHA (ng/mL)	8.000	-0.291	-0.098	0.149	0.577	0.815
AAMA (ng/mL)	2.200	-0.637	-0.486	-0.321	-0.126	0.114
AMCC (ng/mL)	6.260	-0.468	-0.354	-0.176	0.001	0.223
ATCA (ng/mL)	15.000	-0.538	-0.228	0.090	0.330	0.536
BMA (ng/mL)	0.500	-1.637	-1.476	-1.283	-1.043	-0.795
BPMA (ng/mL)	1.200	-2.150	-1.898	-1.526	-1.162	-0.819
CEMA (ng/mL)	6.960	-0.503	-0.339	-0.142	0.050	0.213
CYMA (ng/mL)	0.500	-2.301	-2.095	-1.885	-1.471	-0.534
DHBMA (ng/mL)	5.250	0.225	0.334	0.441	0.547	0.650
HEMA (ng/mL)	0.791	-2.454	-2.258	-2.030	-1.809	-1.586
2HPMA (ng/mL)	5.300	-1.011	-0.836	-0.637	-0.414	-0.179
3HPMA (ng/mL)	13.000	-0.033	0.117	0.288	0.472	0.689
MA (ng/mL)	12.000	-0.241	-0.115	0.022	0.157	0.275
MHBMA3 (ng/mL)	0.600	-1.773	-1.618	-1.443	-1.234	-0.966
PGA (ng/mL)	12.000	-0.004	0.104	0.211	0.325	0.461
HPMMA (ng/mL)	1.700	-0.010	0.088	0.198	0.338	0.607
TT (ng/dL)	0.750	1.199	1.375	1.840	2.606	2.766
E2 (pg/mL)	2.994	1.010	1.253	1.449	1.735	2.135
SHBG (nmol/L)	0.800	1.225	1.404	1.580	1.777	1.977
FAI	-	1.432	1.670	2.245	3.140	3.292
TT/E2	-	-0.722	-0.347	0.828	1.307	1.454

Notes: 2MHA: urinary 2-Methylhippuric acid; 3,4-MHA: urinary 3- and 4-Methylhippuric acid; AAMA: urinary N-Acetyl-S-(2-carbamoylethyl)-L-cysteine; AMCC: urinary N-Acetyl-S-(N-methylcarbamoyl)-L-cysteine; ATCA: urinary 2-Aminothiazoline-4-carboxylic acid; BMA: urinary N-Acetyl-S-(benzyl)-L-cysteine; BPMA: urinary N-Acetyl-

S-(n-propyl)-L-cysteine; CEMA: urinary N-Acetyl-S-(2-carboxyethyl)-L-cysteine; CYMA: urinary N-Acetyl-S-(2-cyanoethyl)-L-cysteine; DHBMA: urinary N-Acetyl-S-(3,4-dihydroxybutyl)-L-cysteine; HEMA: urinary N-Acetyl-S-(2-hydroxyethyl)-L-cysteine; 2HPMA: urinary N-Acetyl-S-(2-hydroxypropyl)-L-cysteine; 3HPMA: urinary N-Acetyl-S-(3-hydroxypropyl)-L-cysteine; MA: urinary Mandelic acid; MHBMA3: urinary N-Acetyl-S-(4-hydroxy-2-butenyl)-L-cysteine; PGA: urinary Phenylglyoxylic acid; HPMMA: urinary N-Acetyl-S-(3-hydroxypropyl-1-methyl)-L-cysteine; TT: testosterone; E_2: estradiol; SHBG: sex hormone-binding globulin; FAI: free androgen index; and TT/E_2: ratio of testosterone to estradiol.

Table S3. Posterior inclusion probabilities (PIPs) in the BKMR model in the NHANES, 2013–2016.

Variable	Exposure	TT	E_2	SHBG	FAI	TT/E_2
	2MHA	0.000	0.638	0.237	0.016	0.041
	3,4-MHA	0.000	0.635	0.009	0.006	0.006
	AAMA	0.103	0.637	0.028	0.010	0.041
	AMCC	0.396	0.759	0.094	0.052	0.039
	ATCA	0.944	1.000	0.016	0.864	0.076
	BMA	0.053	0.818	0.074	0.015	0.080
	BPMA	0.037	0.675	0.029	0.003	0.009
	CEMA	0.083	0.583	0.058	0.015	0.043
Total	CYMA	0.799	0.886	0.000	0.129	0.002
	DHBMA	0.151	0.693	0.014	0.081	0.007
	HEMA	0.030	0.622	0.553	0.152	0.004
	2HPMA	0.267	0.571	0.017	0.109	0.008
	3HPMA	0.169	0.659	0.036	0.093	0.021
	MA	0.184	0.622	0.044	0.086	0.048
	MHBMA3	0.476	0.660	0.084	0.138	0.036
	PGA	0.124	0.773	0.016	0.122	0.036
	HPMMA	0.709	0.603	0.009	0.108	0.441
	2MHA	0.637	0.613	0.404	0.828	0.147
	3,4-MHA	0.528	0.617	0.429	0.803	0.180
	AAMA	0.586	0.732	0.371	0.826	0.463
	AMCC	0.877	0.910	0.493	1.000	0.188
	ATCA	1.000	1.000	0.430	1.000	0.136
	BMA	0.597	0.742	0.432	0.799	0.276
	BPMA	0.872	0.968	0.414	0.955	0.232
	CEMA	0.708	0.666	0.419	0.748	0.505
Male	CYMA	0.966	0.990	0.441	1.000	0.413
	DHBMA	0.680	0.754	0.517	0.741	0.253
	HEMA	0.713	0.903	0.891	0.828	0.223
	2HPMA	0.762	0.709	0.395	0.795	0.243
	3HPMA	0.629	0.658	0.514	0.811	0.393
	MA	0.628	0.600	0.404	0.809	0.273
	MHBMA3	0.852	1.000	0.558	1.000	0.209
	PGA	0.700	0.769	0.507	0.898	0.200
	HPMMA	0.984	0.781	0.551	0.753	0.828
	2MHA	0.524	0.500	0.482	0.688	0.287
	3,4-MHA	0.551	0.456	0.369	0.511	0.232
	AAMA	0.491	0.568	0.395	0.389	0.254
Female	AMCC	0.599	0.514	0.547	0.412	0.261
	ATCA	0.506	0.488	0.473	0.395	0.210
	BMA	0.476	0.637	0.404	0.533	0.521
	BPMA	0.419	0.501	0.344	0.352	0.250

CEMA	0.442	0.482	0.503	0.349	0.240
CYMA	0.723	0.559	0.513	0.342	0.290
DHBMA	0.555	0.557	0.425	0.402	0.311
HEMA	0.460	0.540	0.543	0.469	0.234
2HPMA	0.464	0.440	0.389	0.460	0.285
3HPMA	0.448	0.536	0.412	0.430	0.294
MA	0.522	0.538	0.422	0.431	0.243
MHBMA3	0.465	0.573	0.403	0.461	0.289
PGA	0.523	0.529	0.477	0.414	0.260
HPMMA	0.494	0.567	0.474	0.476	0.270

Notes: 2MHA: urinary 2-Methylhippuric acid; 3,4-MHA: urinary 3- and 4-Methylhippuric acid; AAMA: urinary N-Acetyl-S-(2-carbamoylethyl)-L-cysteine; AMCC: urinary N-Acetyl-S-(N-methylcarbamoyl)-L-cysteine; ATCA: urinary 2-Aminothiazoline-4-carboxylic acid; BMA: urinary N-Acetyl-S-(benzyl)-L-cysteine; BPMA: urinary N-Acetyl-S-(n-propyl)-L-cysteine; CEMA: urinary N-Acetyl-S-(2-carboxyethyl)-L-cysteine; CYMA: urinary N-Acetyl-S-(2-cyanoethyl)-L-cysteine; DHBMA: urinary N-Acetyl-S-(3,4-dihydroxybutyl)-L-cysteine; HEMA: urinary N-Acetyl-S-(2-hydroxyethyl)-L-cysteine; 2HPMA: urinary N-Acetyl-S-(2-hydroxypropyl)-L-cysteine; 3HPMA: urinary N-Acetyl-S-(3-hydroxypropyl)-L-cysteine; MA: urinary Mandelic acid; MHBMA3: urinary N-Acetyl-S-(4-hydroxy-2-butenyl)-L-cysteine; PGA: urinary Phenylglyoxylic acid; HPMMA: urinary N-Acetyl-S-(3-hydroxypropyl-1-methyl)-L-cysteine; TT: testosterone; E_2: estradiol; SHBG: sex hormone-binding globulin; FAI: free androgen index; and TT/E_2: ratio of testosterone to estradiol.

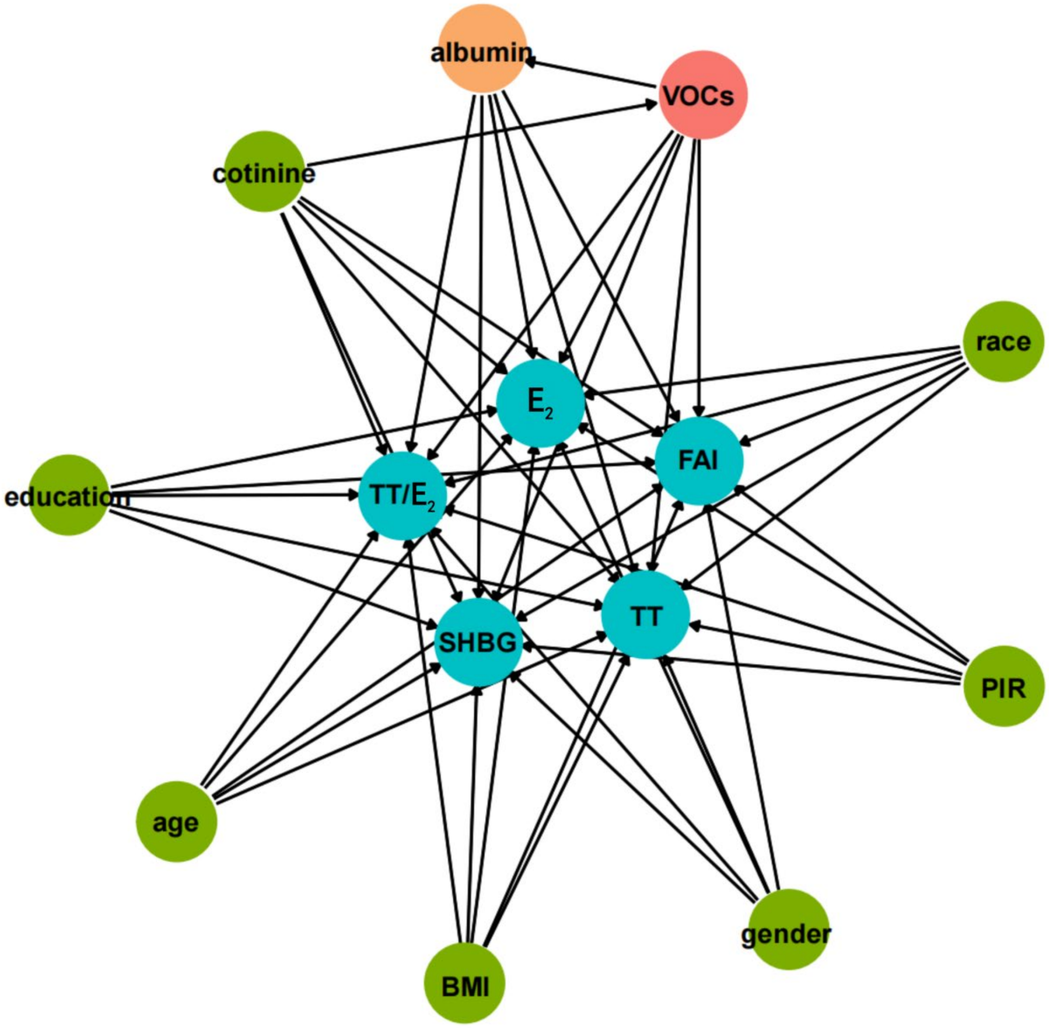

Figure S1. Directed acyclic graph. Notes: VOCs: volatile organic compounds; TT: testosterone; E_2: estradiol; SHBG: sex hormone-binding globulin; FAI: free androgen index; TT/E_2: ratio of testosterone to estradiol; BMI: body mass index; and PIR: the ratio of family income to poverty.

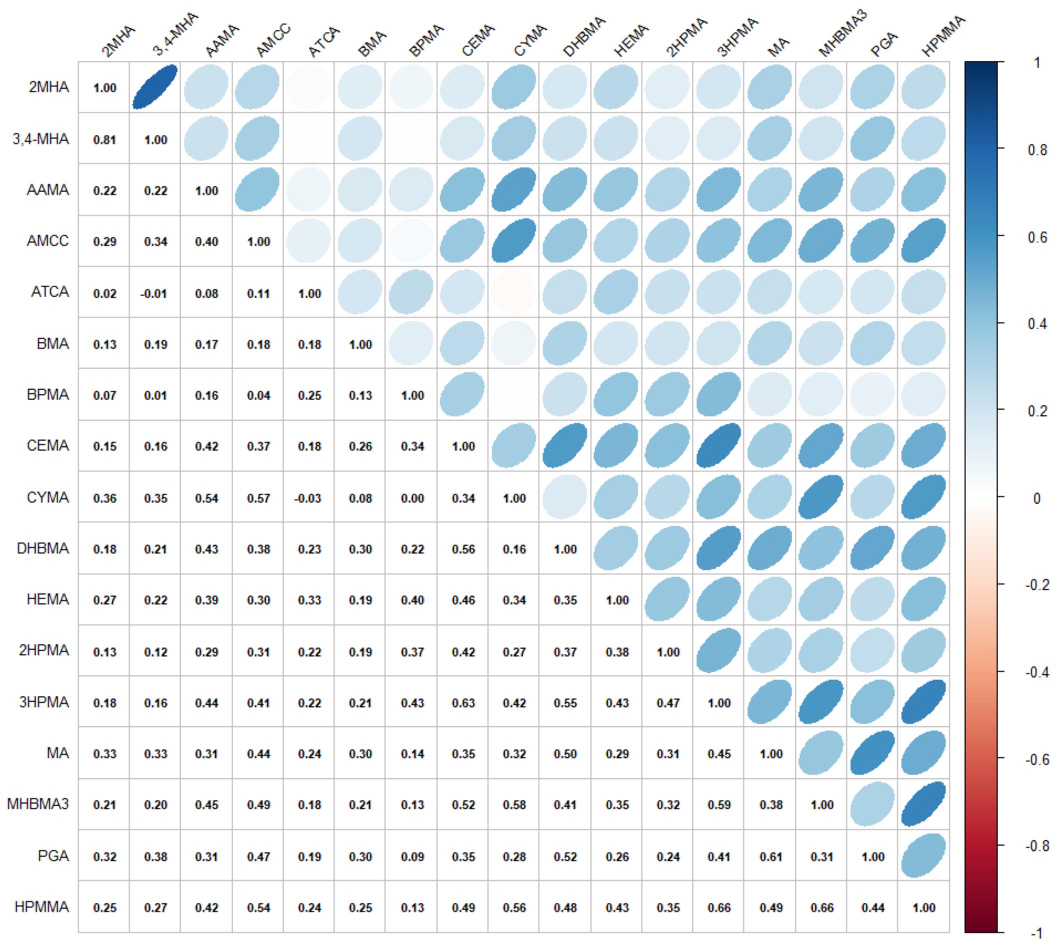

Figure S2. Pearson's correlations between urinary mVOCs. Notes: 2MHA: urinary 2-Methylhippuric acid; 3,4-MHA: urinary 3- and 4-Methylhippuric acid; AAMA: urinary N-Acetyl-S-(2-carbamoylethyl)-L-cysteine; AMCC: urinary N-Acetyl-S-(N-methylcarbamoyl)-L-cysteine; ATCA: urinary 2-Aminothiazoline-4-carboxylic acid; BMA: urinary N-Acetyl-S-(benzyl)-L-cysteine; BPMA: urinary N-Acetyl-S-(n-propyl)-L-cysteine; CEMA: urinary N-Acetyl-S-(2-carboxyethyl)-L-cysteine; CYMA: urinary N-Acetyl-S-(2-cyanoethyl)-L-cysteine; DHBMA: urinary N-Acetyl-S-(3,4-dihydroxybutyl)-L-cysteine; HEMA: urinary N-Acetyl-S-(2-hydroxyethyl)-L-cysteine; 2HPMA: urinary N-Acetyl-S-(2-hydroxypropyl)-L-cysteine; 3HPMA: urinary N-Acetyl-S-(3-hydroxypropyl)-L-cysteine; MA: urinary Mandelic acid; MHBMA3: urinary N-Acetyl-S-(4-hydroxy-2-butenyl)-L-cysteine; PGA: urinary Phenylglyoxylic acid; and HPMMA: urinary N-Acetyl-S-(3-hydroxypropyl-1-methyl)-L-cysteine.

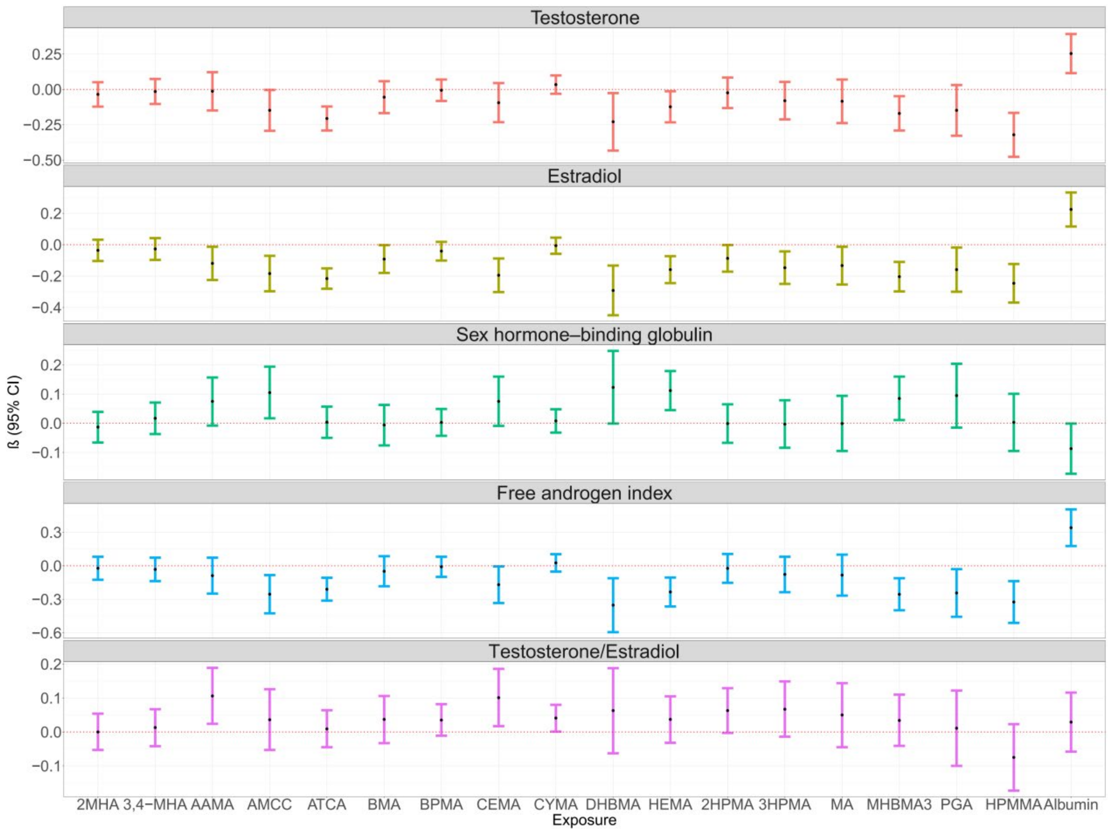

Figure S3. Association of single exposure to mVOCs and serum albumin exposure with sex hormones among male populations in the NHANES, 2013–2016 (N=299). Notes: 2MHA: urinary 2-Methylhippuric acid; 3,4-MHA: urinary 3- and 4-Methylhippuric acid; AAMA: urinary N-Acetyl-S-(2-carbamoylethyl)-L-cysteine; AMCC: urinary N-Acetyl-S-(N-methylcarbamoyl)-L-cysteine; ATCA: urinary 2-Aminothiazoline-4-carboxylic acid; BMA: urinary N-Acetyl-S-(benzyl)-L-cysteine; BPMA: urinary N-Acetyl-S-(n-propyl)-L-cysteine; CEMA: urinary N-Acetyl-S-(2-carboxyethyl)-L-cysteine; CYMA: urinary N-Acetyl-S-(2-cyanoethyl)-L-cysteine; DHBMA: urinary N-Acetyl-S-(3,4-dihydroxybutyl)-L-cysteine; HEMA: urinary N-Acetyl-S-(2-hydroxyethyl)-L-cysteine; 2HPMA: urinary N-Acetyl-S-(2-hydroxypropyl)-L-cysteine; 3HPMA: urinary N-Acetyl-S-(3-hydroxypropyl)-L-cysteine; MA: urinary Mandelic acid; MHBMA3: urinary N-Acetyl-S-(4-hydroxy-2-butenyl)-L-cysteine; PGA: urinary Phenylglyoxylic acid; and HPMMA: urinary N-Acetyl-S-(3-hydroxypropyl-1-methyl)-L-cysteine. Adjusted for age, race/ethnicity, education level, BMI, serum cotinine concentration, and the ratio of family income to poverty.

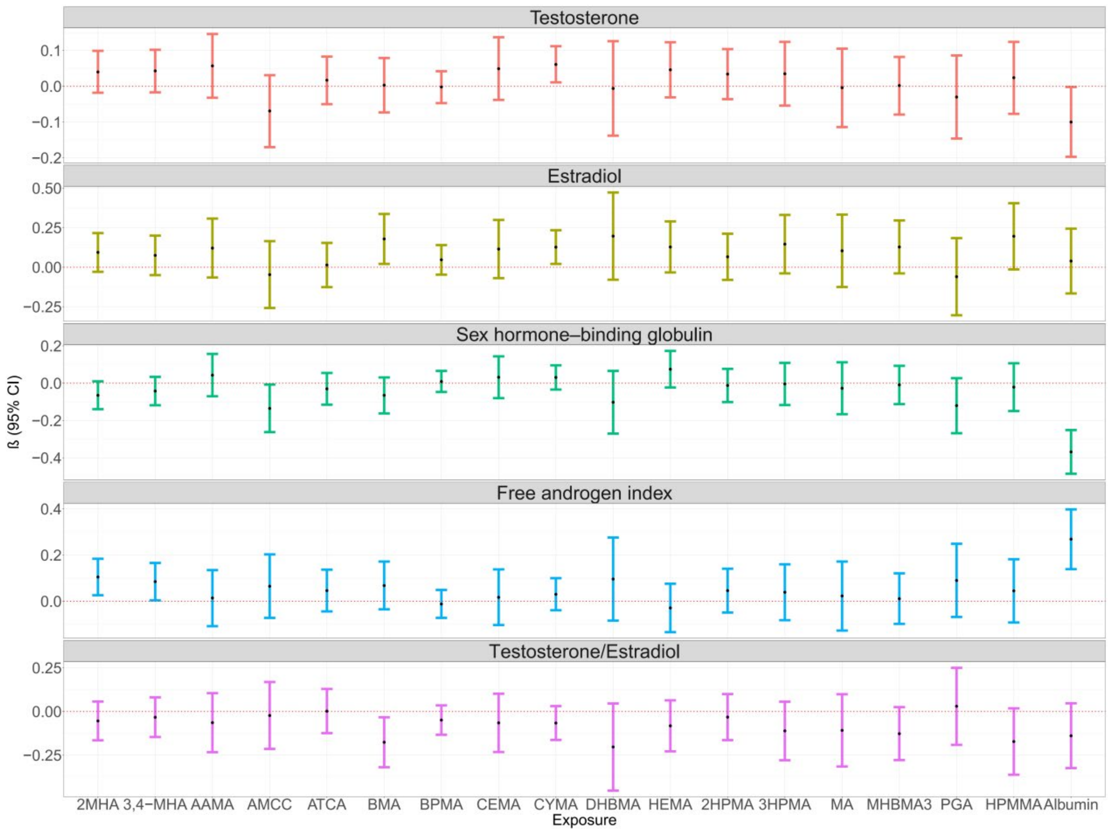

Figure S4. Association of single exposure to mVOCs and serum albumin exposure with sex hormones among female populations in the NHANES, 2013–2016 (N=285). Notes: 2MHA: urinary 2-Methylhippuric acid; 3,4-MHA: urinary 3- and 4-Methylhippuric acid; AAMA: urinary N-Acetyl-S-(2-carbamoylethyl)-L-cysteine; AMCC: urinary N-Acetyl-S-(N-methylcarbamoyl)-L-cysteine; ATCA: urinary 2-Aminothiazoline-4-carboxylic acid; BMA: urinary N-Acetyl-S-(benzyl)-L-cysteine; BPMA: urinary N-Acetyl-S-(n-propyl)-L-cysteine; CEMA: urinary N-Acetyl-S-(2-carboxyethyl)-L-cysteine; CYMA: urinary N-Acetyl-S-(2-cyanoethyl)-L-cysteine; DHBMA: urinary N-Acetyl-S-(3,4-dihydroxybutyl)-L-cysteine; HEMA: urinary N-Acetyl-S-(2-hydroxyethyl)-L-cysteine; 2HPMA: urinary N-Acetyl-S-(2-hydroxypropyl)-L-cysteine; 3HPMA: urinary N-Acetyl-S-(3-hydroxypropyl)-L-cysteine; MA: urinary Mandelic acid; MHBMA3: urinary N-Acetyl-S-(4-hydroxy-2-butenyl)-L-cysteine; PGA: urinary Phenylglyoxylic acid; and HPMMA: urinary N-Acetyl-S-(3-hydroxypropyl-1-methyl)-L-cysteine. Adjusted for age, race/ethnicity, education level, BMI, serum cotinine concentration, and the ratio of family income to poverty.

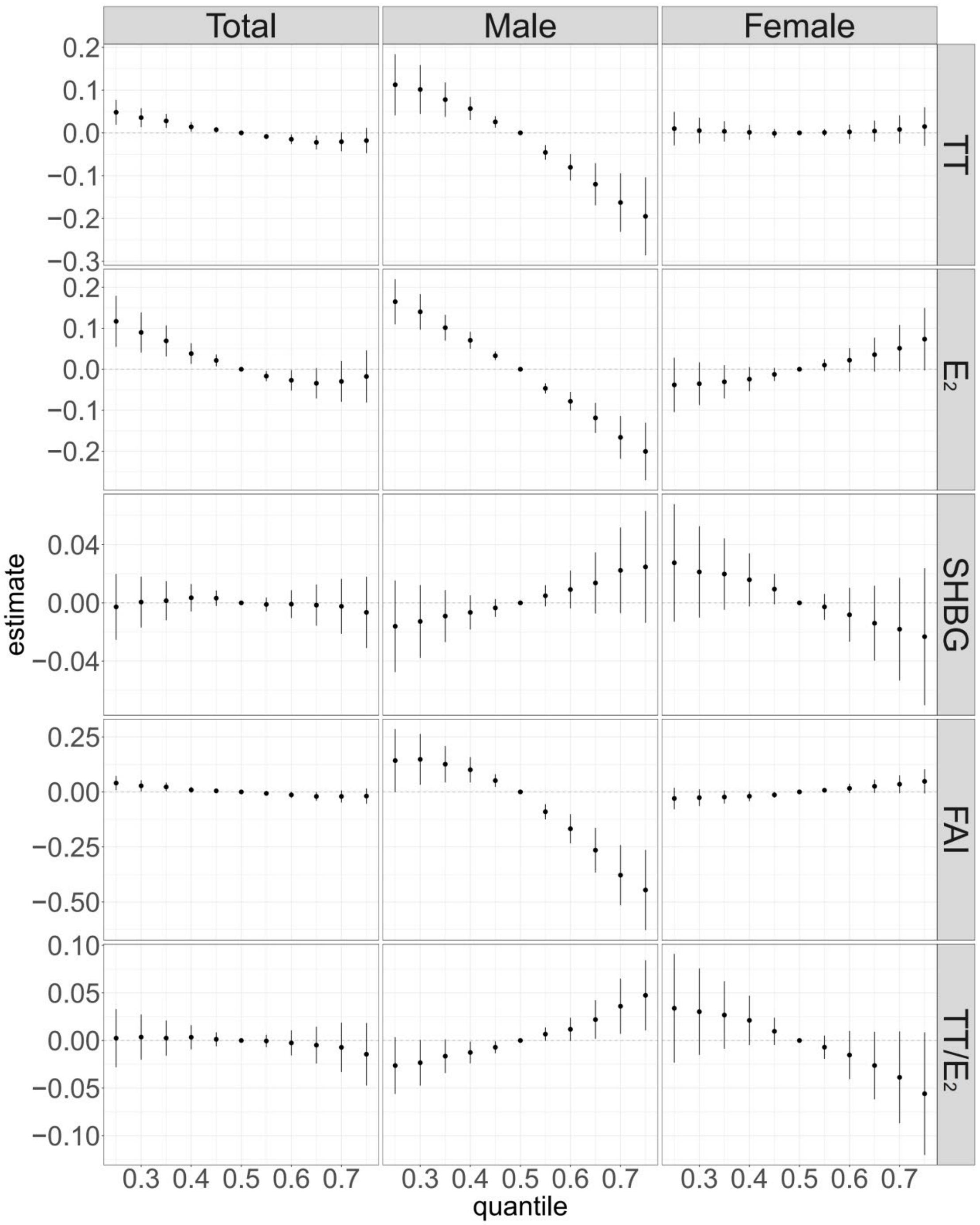

Figure S5. The effects of mVOC mixture exposure on TT, E2, SHBG, FAI, and TT/E2 by the BKMR models. Notes: The ordinate showed the estimated change in the sex hormones when all the VOC chemicals were at particular percentiles compared to when they were at their 50th percentile. TT: testosterone; E_2: estradiol; SHBG: sex hormone-binding globulin; FAI: free androgen index; and TT/E_2: ratio of testosterone to estradiol.

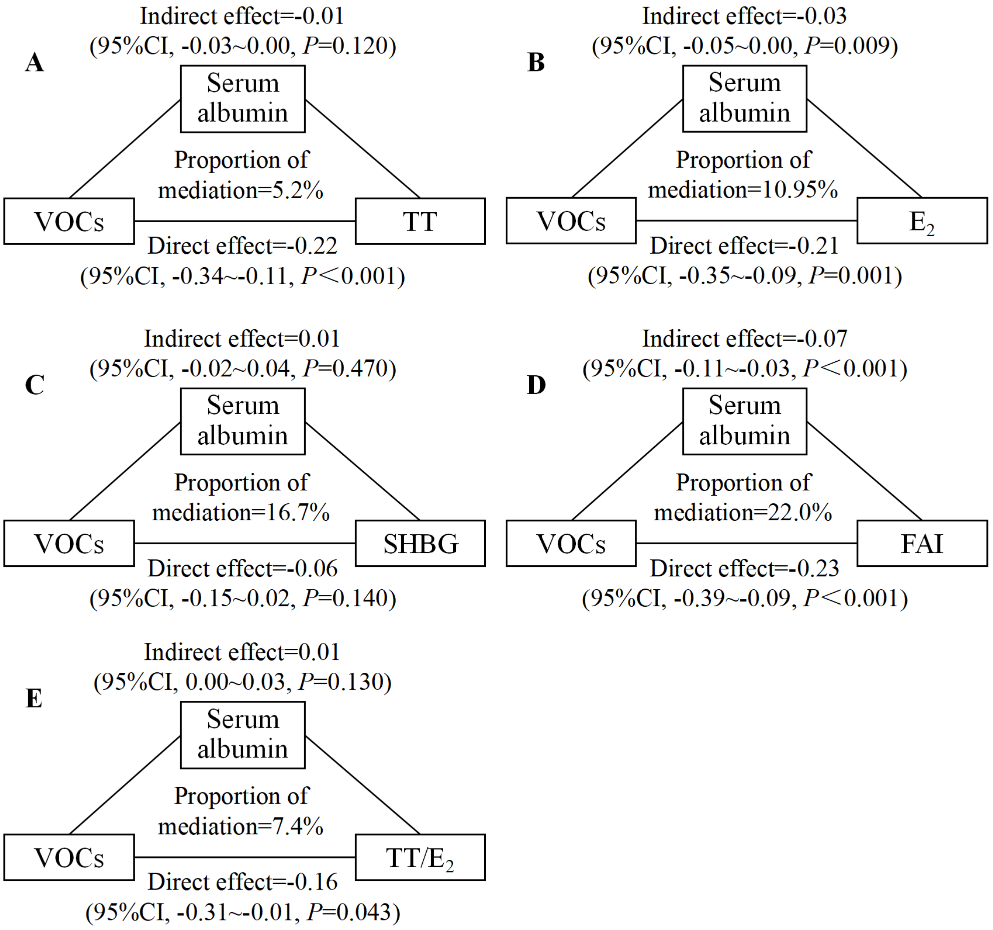

Figure S6. Estimated proportions of associations between mixed exposure to mVOCs and TT, E2, SHBG, FAI, and TT/E2 mediated by serum albumin (N=584). Notes: CI: confidence interval; VOCs: volatile organic compounds; TT: testosterone; E_2: estradiol; SHBG: sex hormone-binding globulin; FAI: free androgen index; and TT/E_2: ratio of testosterone to estradiol. Adjusted for gender, age, race/ethnicity, education level, BMI, serum cotinine concentration, and the ratio of family income to poverty.
